# Enhancing Consultation Efficiency Through Medical Informatics: A Scalable Field Clinic Model for the Pandemic Response in Taiwan

**DOI:** 10.3390/healthcare13131514

**Published:** 2025-06-25

**Authors:** Chun-Li Wang, Chung-Fu Li, Chiann-Yi Hsu, Pi-Shan Hsu

**Affiliations:** 1 Health Management Center, Taichung Veterans General Hospital, Taichung 407219, Taiwan; chun_li@vghtc.gov.tw; 2 Department of Family Medicine, Taichung Veterans General Hospital, Taichung 407219, Taiwan; jackie.li2582@vghtc.gov.tw; 3 Institute of Medicine, Chung Shan Medical University, Taichung 402306, Taiwan; 4 Biostatistics Group, Department of Medical Research, Taichung Veterans General Hospital, Taichung 407219, Taiwan; chiann@vghtc.gov.tw; 5 Graduate Institute of Microbiology and Public Health, College of Veterinary Medicine, National Chung-Hsing University, Taichung 402202, Taiwan

**Keywords:** COVID-19, field clinic, health informatics, workflow optimization, electronic health record, public health emergency

## Abstract

**Background:** During the COVID-19 pandemic, a high-volume field clinic was rapidly established in Taichung, Taiwan, to manage patients with mild symptoms and reduce hospital burden. To streamline workflow and support timely care, a tailored medical informatics system was developed and implemented midway through clinic operations. **Methods:** We conducted a retrospective observational study analyzing data from 8287 patients who visited the clinic between 20 May and 4 June 2022. Patients were divided into two groups based on whether they received care before or after the informatics system was introduced (28 May). The primary outcomes included consultation volume, physician workload distribution, and operational efficiency during peak hours. A secondary analysis examined the subgroup receiving antiviral prescriptions. **Results:** After system implementation, the total number of consultations during peak hours increased significantly (from 138.6 to 199.0, a 43.5% increase; *p* = 0.001), along with the average number of consultations per physician (from 12.3 to 22.5, an 83% increase; *p* = 0.003). Similar trends were observed in the subgroup receiving antiviral therapy, despite the complexity of prescribing decisions. These prescribing trends suggest improved identification of high-risk patients and more timely antiviral initiation, which are critical for reducing disease progression and preventing hospitalization. **Conclusions:** The integration of a targeted medical informatics system significantly improved consultation efficiency and workload equity in a field clinic setting. This experience highlights a scalable model for digitally enhanced, rapid-response outpatient care during public health emergencies.

## 1. Introduction

Severe Acute Respiratory Syndrome Coronavirus 2 (SARS-CoV-2), the causative agent of Coronavirus Disease 2019 (COVID-19), emerged in late 2019 and rapidly escalated into a global pandemic. By December 2024, the World Health Organization (WHO) reported over 776 million confirmed cases worldwide, including more than 7 million deaths [1].

While many countries experienced repeated waves of infection and increasing healthcare burdens from early 2020 onward, Taiwan initially maintained low case numbers due to strict border control and public health containment strategies. However, this early success also meant that the healthcare system had limited exposure to large-scale community transmission. When cases surged in May 2022, the outpatient care sector was quickly overwhelmed, exposing key operational vulnerabilities and highlighting the urgent need for scalable outpatient triage solutions. On 27 May 2022, Taiwan reported a peak of 94,855 daily COVID-19 cases, with cumulative cases surpassing 1.7 million. This rapid escalation underscored the urgent need for scalable and efficient healthcare responses to manage the surge in demand.

To alleviate the strain on healthcare systems during the COVID-19 pandemic, temporary care sites were rapidly established to manage patients with mild symptoms. These facilities provided essential services such as testing, clinical evaluation, and medication prescription, thereby easing the burden on hospitals and preserving capacity for critically ill patients [2,3,4,5]. Many such models were deployed as field hospitals, primarily intended for inpatient decompression, quarantine, or surge capacity [3,6,7,8]. While field hospitals have been widely documented—particularly in China, South Africa, and UN missions—for their contributions to inpatient COVID-19 management, comparatively fewer studies have focused on outpatient-based field clinics designed for the rapid triage and treatment of mildly symptomatic patients.

Health informatics rapidly emerged as a key strategy during the COVID-19 pandemic, enabling more efficient and responsive care delivery through enhanced workflows, reduced errors, and real-time clinical decision support [9,10,11]. Informatics approaches—such as electronic health record (EHR) optimization, telehealth integration, and care coordination platforms—have been widely adopted to improve healthcare system adaptability and resilience under crisis conditions [10,11,12,13]. These technologies have shown potential to enhance both efficiency and accuracy in patient diagnosis and treatment [12,14,15,16]. However, few studies have provided empirical evidence demonstrating measurable improvements in real-world consultation efficiency, particularly in high-demand outpatient environments such as field clinics. This gap remains critical given the operational challenges posed by pandemic surges and the urgent need for scalable, informatics-driven care models [17,18].

To address the current lack of empirical evidence on real-world consultation efficiency, this study aims to evaluate the operational impact of a novel medical informatics system implemented at a high-volume outpatient field clinic during Taiwan’s COVID-19 surge, where improving throughput was essential to maintaining equitable and timely access to care amid overwhelming demand. In such constrained settings, operational efficiency directly influences not only patient flow but also resource allocation, wait times, and the ability to deliver early antiviral therapy to high-risk individuals. The experience gained may serve as a reference for implementing scalable, informatics-supported outpatient care models in future emergency settings.

## 2. Materials and Methods

### 2.1. Field Clinic Setup and Patient Flow

A field clinic was established at Taichung Central Park, a large public park covering approximately 24 hectares (240,000 square meters). The multidisciplinary team involved in the field clinic consisted of 71 physicians, 203 nurses, and 40 additional healthcare personnel, including medical laboratory scientists, technicians, pharmacists, administrative staff, security guards, and volunteers. The clinic operated two daily shifts, 9:00 AM to 12:30 PM and 1:30 PM to 5:00 PM, and it remained active from 20 May to 4 June 2022.

**Figure 1:**
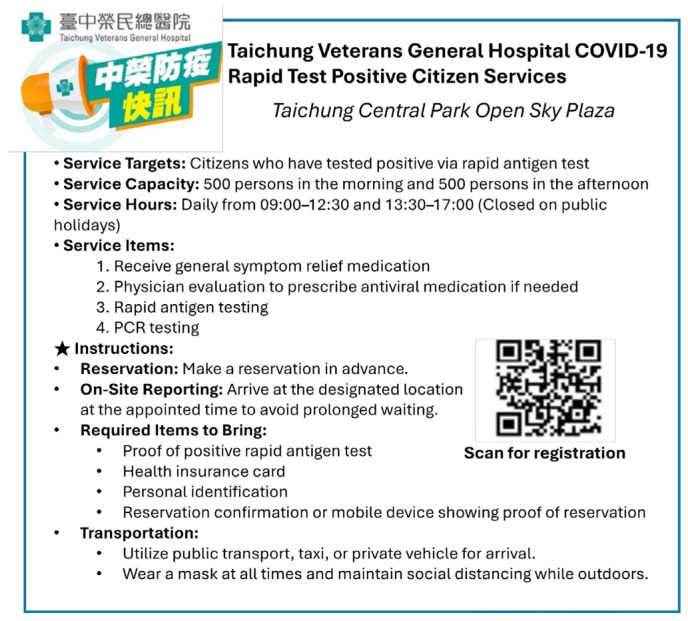
Social media announcement of field clinic services and registration method. A post from Taichung Veterans General Hospital’s official social media platform promoting field clinic services. The image details the clinic’s services, including polymerase chain reaction (PCR) testing, physician consultation, and prescriptions for symptomatic relief and antiviral medications. It also provides information about the clinic’s location and operating hours. The Quick Response (QR) code directs users to an online pre-registration platform, allowing patients to register in advance for streamlined clinic access. The original image includes the Chinese terms “臺中榮民總醫院” (Taichung Veterans General Hospital) and “中榮防疫快訊” (COVID-19 Alerts by Taichung Veterans General Hospital), which are names of the institution and its public health information series, respectively.

Patients who received a positive result from a self-administered COVID-19 rapid antigen test were eligible to register in advance through the clinic’s website or by scanning a Quick Response (QR) code promoted on the official social media platforms of the Taichung City Government and Taichung Veterans General Hospital (Figure 1).

Upon arrival, patients were triaged into one of two primary zones: the polymerase chain reaction (PCR) testing cabin or the physician consultation zone. Individuals with a confirmed positive result from a home-based rapid test were directed straight to the consultation zone. In contrast, patients presenting with COVID-19-related symptoms but without prior testing were referred to the PCR testing cabin for diagnostic confirmation before further evaluation.

Following PCR testing, patient flow proceeded as follows:

1. PCR-positive individuals were routed to the consultation zone for medical evaluation and, if indicated, antiviral prescription.
2. PCR-negative individuals were discharged without further clinical assessment.

**Figure 2:**
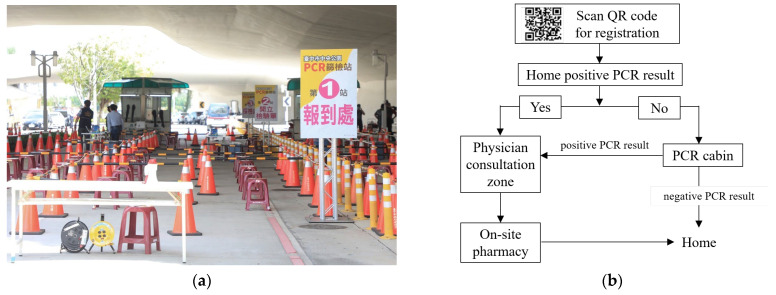
Overview of the COVID-19 field clinic at Taichung Central Park. (**a**) On-site photograph showing the actual layout of the field clinic, including queue arrangements under temporary tents, medical staff in protective equipment, signage designating service areas, and patient routing during peak operating hours. (**b**) Schematic diagram of patient flow and triage pathways. Patients were routed based on prior testing status and PCR results. Each clinical zone included four designated queues: one for elderly individuals (aged ≥ 65 years), one for parents with children, and two for the general adult population. (**a**) includes the Chinese term “第1站報到處,” which translates to “Station 1: Registration,” indicating the check-in point at the start of patient flow.

To ensure safety and improve operational efficiency, each service zone was further divided into four designated queues based on patient characteristics: one for elderly individuals (aged ≥ 65 years), one for parents accompanied by children, and two for the public. This structured queuing system minimized crowding, reduced waiting times, and facilitated orderly patient flow throughout the clinic. After completing the consultation, patients proceeded to the on-site pharmacy area to collect their prescribed medications before exiting the facility. Figure 2 presents both the physical layout and patient flow design of the COVID-19 field clinic, highlighting key features of triage, queue management, and spatial organization.

### 2.2. Medication Administration and Treatment Protocols

A standardized range of medications was provided to relieve common COVID-19 symptoms, including analgesics, antihistamines, and decongestants. In addition, two oral antiviral agents—ritonavir-boosted nirmatrelvir (Paxlovid) and molnupiravir (Lagevrio)—were available, with treatment selection guided by each patient’s clinical presentation and eligibility.

Antiviral therapy was considered for patients who presented within five days of symptom onset and met the risk criteria for progression to moderate or severe disease, as defined by the U.S. Centers for Disease Control and Prevention (CDC; Table S1). These prescriptions required individualized clinical judgment, including an assessment of renal function, comorbidities, and potential drug–drug interactions. Dosages were adjusted accordingly to ensure safety and effectiveness. All prescribed medications were reviewed and dispensed by on-site pharmacists, who also provided patient counseling and education regarding proper use, potential side effects, and follow-up instructions.

### 2.3. Development and Implementation of the Medical Informatics Program System

To enhance workflow efficiency and ensure safe, accurate prescribing, we developed a medical informatics system specifically designed to meet the operational needs of the COVID-19 field clinic. One of its core features is the automatic consolidation of frequently prescribed medications—previously selected manually—into predefined medication packages, dynamically generated based on usage patterns in the preceding days.

The system was developed to support clinicians through a structured, five-step workflow that integrates clinical data, national health insurance records, and evidence-based treatment logic to optimize decision-making and medication management.

Step 1. Patient Identification: The clinical workflow is initiated by inserting the National Health Insurance (NHI) card to verify patient identity.

Step 2. Clinical Assessment and Documentation: Physicians evaluate the patient’s clinical condition, including age group (≥8 or <8 years), body weight, and rapid antigen test results. The system then auto-generates an editable electronic medical record using structured templates, which physicians can freely modify or expand according to clinical judgment.

Step 3. Case Reporting: For patients who test positive via a rapid antigen test, physicians report the case to the National Health Insurance Administration (NHIA) by selecting the test date and entering the patient’s contact information. This step ensures that the case is officially documented for public health reporting and included in national surveillance data.

Step 4. Review of Cloud-Based Health Records: Physicians access the NHIA cloud system to retrieve each patient’s medical history, current medications, and renal function. This information supports clinical safety and antiviral eligibility assessment.

**Figure 3:**
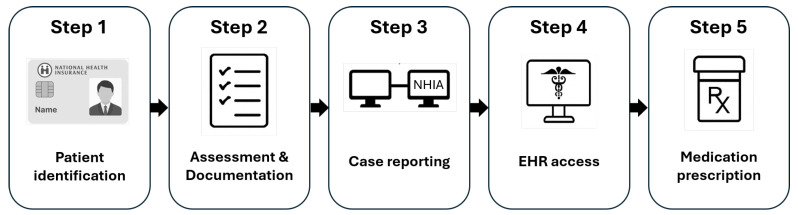
Physician interface of the five-step medical informatics system used to streamline COVID-19 field clinic workflows, from National Health Insurance (NHI) card identification to medication prescription.

Step 5. Medication Prescription: Physicians prescribe structured medication bundles tailored to common COVID-19 symptoms. For antiviral therapy, the system recommends the most suitable agent—ritonavir-boosted nirmatrelvir (Paxlovid) or molnupiravir (Lagevrio)—based on the patient’s clinical history and renal function while preserving physician discretion for final decisions. The user interface of the implemented informatics program is illustrated in Figure 3.

### 2.4. Data Source and Study Design

Medical records were retrospectively reviewed to identify all patients who tested positive for COVID-19 via a rapid self-test performed at home and subsequently visited the field clinic at Taichung Central Park between 20 May and 4 June 2022.

To assess the impact of the newly implemented medical informatics system on consultation and treatment efficiency, patients were categorized into two groups based on the system’s go-live date of 28 May 2022. Only patients who visited the clinic during the peak operational hours of 10:00 AM to 12:00 PM were included in the final analysis. Visits outside this time window were excluded due to lower patient volumes and greater variability in clinical operations.

To evaluate consultation efficiency, three indicators were calculated for the study period: the average number of consultations per physician, the maximum number of consultations recorded by any physician, and the minimum number of consultations. For the secondary analysis, patients were further stratified according to whether they received antiviral medications during their visit, as these prescriptions involved more complex clinical decision-making.

Data collected included patients’ sex, age, antiviral medication usage, and baseline vital signs, including oxygen saturation on room air. For each clinic day, the number of physicians on duty and the total number of patient consultations during peak hours (10:00 AM to 12:00 PM) were also recorded. Physicians were classified by seniority (resident or attending) and specialty (internal medicine, surgery, or other). All medical records were extracted under de-identification protocols by the Clinical Informatics Research & Development Center (CIRDC). The study protocol was approved by the Institutional Review Board of Taichung Veterans General Hospital (IRB number CE22326A#1, with approval granted on 26 September 2022).

All data used in this study were retrieved from structured electronic administrative databases maintained by the field clinic. These included standardized variables such as visit timestamps, physician identifiers, and prescription records. Data extraction was performed using pre-defined scripts applied to structured electronic databases, ensuring consistency and reproducibility, and the results were independently verified by a second research team member to ensure accuracy and completeness. As no manual chart review or subjective interpretation was involved, inter-rater reliability assessment was not applicable.

### 2.5. Statistical Analysis

Associations between categorical variables and the two study periods were assessed using the chi-square test, while comparisons of continuous variables were performed using the Mann–Whitney U test. The normality of continuous variables was assessed using the Kolmogorov–Smirnov test. As the metrics did not follow a normal distribution (*p* < 0.05), non-parametric testing was deemed appropriate.

Continuous data are presented as means ± standard deviations, and categorical variables are reported as frequencies and percentages. A two-tailed *p*-value of <0.05 was considered statistically significant. All statistical analyses were conducted using SPSS software, version 22.0 (IBM Corp., Armonk, NY, USA).

## 3. Results

### 3.1. Patient Demographics and Clinical Characteristics

As shown in Table 1, the average number of new daily COVID-19 cases in Taichung increased slightly during the post-implementation period, although this change was not statistically significant (*p* = 0.372). Similarly, the average number of patients visiting the field clinic remained stable before and after implementation (469 vs. 454 per day, *p* = 0.762), suggesting comparable levels of service demand across the study period. On peak days, the clinic accommodated more than 600 patients, highlighting its substantial consultation capacity within limited daily operating hours.

A total of 8287 patients visited the field clinic between 20 May and 4 June 2022, with 3749 attending before and 4538 attending after the implementation of the medical informatics system. There were no significant differences in age (39.73 ± 18.64 years, *p* = 0.981) or sex distribution (52.1% male, *p* = 0.058) between the two groups. The proportion of patients receiving antiviral medications was significantly higher after system implementation (15.3% vs. 9.6%, *p* < 0.001), driven primarily by an increase in Paxlovid prescriptions (14.4% vs. 8.9%, *p* < 0.001). In contrast, the use of Molnupiravir remained low and comparable across both groups (0.9% vs. 0.8%, *p* = 0.455). Significant differences were also observed in physician composition. The proportion of attending physicians increased markedly (91.2% vs. 65.7%, *p* < 0.001), while resident physician participation declined. Additionally, a shift toward surgical specialties was noted during the post-implementation period (42.7% vs. 31.1%, *p* < 0.001) (Table 2).

### 3.2. Consultation Efficiency During Peak Hours

**Figure 4:**
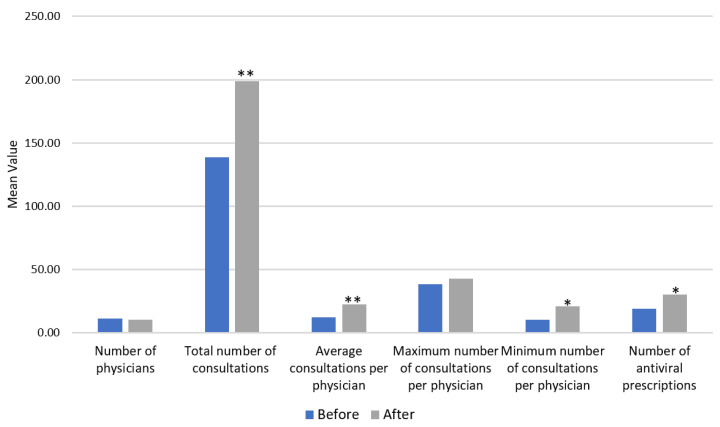
Consultation outcomes before and after the implementation of the medical informatics system during peak hours. Asterisks indicate statistical significance (* *p* < 0.05, ** *p* < 0.01).

The primary outcome analysis focused on the consultation metrics during the 10:00 AM–12:00 PM period. Following the implementation of the medical informatics system, the number of antiviral prescriptions increased significantly (30.00 vs. 19.00, *p* = 0.046). The total number of patient consultations also rose markedly (199.00 vs. 138.63, *p* = 0.001), accompanied by a significant increase in the average number of consultations per physician (22.51 vs. 12.28, *p* = 0.003). Although the maximum number of consultations per physician did not differ significantly (*p* = 0.370), the minimum number increased significantly (21.20 vs. 10.38, *p* = 0.015), indicating a more balanced distribution of the workload (Table 3 and Figure 4).

### 3.3. Subgroup Analysis Based on Antiviral Prescription

In the subgroup of patients who received antiviral medications, the total number of consultations significantly increased after system implementation (30.00 vs. 19.00, *p* = 0.046). Although the number of physicians increased (5.60 vs. 4.63), this difference was not statistically significant (*p* = 0.102). Other efficiency indicators, including the average, maximum, and minimum number of consultations per physician, showed non-significant trends toward improvement. Among patients who did not receive antiviral medications, the total number of consultations also increased significantly (169.00 vs. 119.63, *p* = 0.007), accompanied by a significant rise in the average number of consultations per physician (27.18 vs. 20.82, *p* = 0.032) (Table 4).

## 4. Discussion

Our study demonstrates that a temporary COVID-19 field clinic in Taiwan was able to provide high-volume outpatient services within a condensed time frame, achieving a total of over 8000 patient visits in just 16 days. This level of service reflects not only the urgency of care required during the surge but also the operational feasibility of a well-organized field setting. Importantly, the implementation of the newly developed medical informatics system led to a marked improvement in consultation efficiency. During peak hours, the total number of consultations rose by 43.5% (from 138.6 to 199.0, *p* = 0.001), and the average number of consultations per physician increased by 83% (from 12.3 to 22.5, *p* = 0.003). The minimum number of consultations per physician nearly doubled (from 10.38 to 21.20, *p* = 0.015), indicating a more equitable distribution of the workload across the clinical team.

Although physicians were able to manage more patients and issue a higher number of antiviral prescriptions after system implementation, a subgroup analysis revealed more modest efficiency gains among those receiving antivirals. This observation aligns with prior findings that digital tools can enhance clinical efficiency, even under high-pressure conditions [19,20,21]. The relatively smaller effect in this subgroup likely reflects the greater clinical complexity of these cases, which require additional assessments. While these improvements in consultation throughput are encouraging, we did not assess whether such gains compromised care quality—such as missed diagnoses, reduced interaction times, or patient satisfaction. Assessing patient-centered outcomes will be essential to ensure that operational efficiency does not come at the expense of safety or care experience.

Notably, the proportion of patients prescribed antiviral therapy increased significantly from 9.6% to 15.3% (*p* < 0.001), primarily due to a rise in Paxlovid prescriptions (from 8.9% to 14.4%). This increase may be attributed to several contextual factors, including real-time eligibility prompts embedded in the system that supported timely prescribing decisions, growing public awareness of treatment availability, and a national guideline revision announced on 24 May 2022, which expanded Paxlovid eligibility to include postpartum women.

Furthermore, the shift toward a higher proportion of attending physicians (91.2% post-implementation) may have partially influenced consultation efficiency, as senior clinicians often complete evaluations more rapidly than residents. This staffing change represents a potential confounding factor and should be considered when interpreting the magnitude of the system-driven improvements.

### 4.1. Comparison with International Field Clinic Models

Outpatient service models during the COVID-19 pandemic varied significantly across countries. Taiwan’s field clinic model stood out for its integration of clinical operations with digital tools, including pre-registration, triage zoning, and real-time data flow. Unlike many settings that relied on manual workflows [4], this approach improved efficiency, reduced crowding, and minimized administrative burden. Notably, integration with the National Health Insurance Administration (NHIA) enabled real-time access that supported safe antiviral prescribing by reducing drug–drug interactions and avoiding duplicate treatment.

Detailed structural planning, such as that implemented in Taiwan’s field clinic model, is rarely described in field-based outpatient services elsewhere. In the United States, mobile clinics served important roles in reaching underserved populations, but they often lacked diagnostic capacity, pharmaceutical inventory, and workflow integration—as seen in community-based efforts in Baltimore that combined medical and social outreach [5,17]. Compared to Taiwan’s model, these clinics typically operated with a limited digital infrastructure, lacked centralized prescribing protocols, and were not connected to national health systems for real-time clinical decision-making. Similarly, in South Africa, field hospitals functioned primarily as inpatient decompression units with limited adaptability for rapid outpatient care, even in models designed for flexible surge responses [18]. In many countries, patients with mild COVID-19 symptoms were advised to remain at home. While this approach aimed to preserve hospital resources, it has been associated with increased levels of anxiety, depression, and other mental health issues, as well as delayed access to appropriate care [22,23,24]. Although such efforts reflect meaningful attempts to expand care beyond traditional hospitals, Taiwan’s model distinguishes itself through nationwide coordination, affordable access, and seamless digital integration. This interoperable framework enabled both equitable coverage and streamlined clinical operations. As health systems prepare for future health emergencies, Taiwan’s experience could serve as a potential reference, particularly for settings aiming to rapidly deploy outpatient services under resource constraints.

### 4.2. Role of Health Informatics System Design

The success of this field clinic was closely tied to the design and deployment of its health informatics system. Tailored for time-sensitive outpatient settings, the system emphasized usability and efficiency without compromising accuracy. It featured a structured five-step workflow, integrated clinical logic for medication decisions, and the automated bundling of frequently used treatments. Built to handle surges in patient volume, the platform reduced delays through streamlined processes and standardized support tools. These functions were especially critical for managing patients at high risk of severe disease [25,26]. Physicians could quickly assess eligibility and initiate antiviral therapy using predefined protocols aligned with patient age and clinical risk factors [26], thereby reducing administrative burden and enhancing prescribing safety. Previous studies have emphasized the strategic importance of informatics in pandemic care, including its role in enhancing health system responsiveness [9], accelerating workflow adaptation and order set deployment [10], and supporting digital decision-making through telehealth and data dashboards [12]. Digital health frameworks have been recognized as pivotal in reshaping healthcare delivery during the COVID-19 pandemic, particularly through their capacity to support system redesign, data integration, and remote services across diverse settings [27]. In the African context, national-level digital infrastructure was shown to facilitate rapid public health responses, highlighting the importance of coordinated digital systems in resource-constrained environments [28].

While these studies underscore the potential of digital health, they primarily focus on system design, governance, or theoretical frameworks. In contrast, our study provides operational evidence from a real-world outpatient clinic operating in a high-demand environment. By quantifying changes in consultation capacity and physician workload distribution, we demonstrate how a field-deployed informatics system can enhance clinical efficiency under pressure.

### 4.3. Telemedicine Integration and Future Directions

In parallel with the development of field-based informatics systems, the pandemic accelerated the adoption of telemedicine, which addressed barriers related to access, infection control, and continuity of care in restricted environments [29,30]. This hybrid model has proven feasible in real-world studies, including remote follow-up for orthopedic recovery and outpatient triage via mobile messaging platforms [31,32,33].

Taiwan’s model represents a rare example of a field-based outpatient service that achieved both clinical capacity and organizational coherence. The integration of multidisciplinary care teams, onsite medication dispensing, and real-time informatics enabled sustained, high-volume operations with minimal delays. This alignment between system design and frontline execution likely supported the clinic’s ability to maintain safety and responsiveness under evolving pandemic pressures. To the best of our knowledge, few studies have examined the use of field clinics for the outpatient management of COVID-19 patients with mild-to-moderate symptoms, particularly in relation to quantifiable improvements in efficiency and physician workload distribution.

This study has several limitations. First, it was based on a single-center experience and adopted a retrospective observational design, which limits its generalizability and precludes causal inference. The inclusion of multi-center data in future studies would enhance external validity and allow for comparison across different care environments.

Second, the observed improvements in service efficiency may also have been influenced by unmeasured confounding factors such as staffing fluctuations, learning curves, or changes in patient behavior during the study period. In addition, factors such as physician fatigue, shift schedules, or cumulative workload may have also played a role. For example, attending physicians working multiple consecutive days may experience fatigue that affects performance or, conversely, become more proficient over time. These effects may not be evenly distributed across all providers and could have biased the observed efficiency gains.

Although we considered conducting sensitivity analyses, the nature of the data precluded stratified or scenario-based testing. Nonetheless, potential temporal bias was partially mitigated by using a fixed implementation date and the relative stability of city-wide COVID-19 case volumes during the study period. Future studies should incorporate more granular operational data, such as provider-specific caseloads, consultation durations, and shift rosters, to better distinguish system-level effects from variations in clinical practice or staff dynamics.

Third, the informatics system was built upon Taiwan’s national health insurance infrastructure, which may not be readily transferable to healthcare environments with fragmented coverage or limited digital maturity. In particular, the system’s integration with a centralized electronic health record and insurance platform enabled real-time access to medical histories and prescription eligibility, which may not be feasible in settings without a comparable infrastructure. Therefore, similar efficiency gains may not be achievable in countries lacking unified health data systems.

Forth, we did not collect data on patient outcomes beyond clinical encounters such as symptom resolution, treatment adherence, or subsequent hospitalization. This limits our ability to evaluate the downstream clinical effectiveness of the care provided. Longitudinal and multi-site studies are warranted to evaluate both the clinical impact and generalizability of this outpatient care model.

Looking ahead, the findings of this study support the inclusion of field-based clinics, augmented by scalable informatics tools, as a core strategy in national pandemic preparedness. As novel pathogens continue to emerge and strain healthcare capacity, the ability to rapidly deploy outpatient care models that are digitally enabled, patient-accessible, and resource-efficient will be essential. Future systems should aim to be modular, interoperable, and adaptable to diverse settings with varying levels of digital readiness. The integration of real-time dashboards, clinical decision support, and remote monitoring may further enhance responsiveness and continuity of care.

## 5. Conclusions

This study demonstrates that a cloud-connected medical informatics system significantly improved consultation efficiency and workload balance in a high-volume COVID-19 field clinic in Taiwan. By streamlining triage, prescribing, and access to health records, the system enabled scalable and safe outpatient care under pandemic conditions. Taiwan’s field clinic model, featuring rapid deployment and digital integration, may serve as a reference for countries seeking to strengthen outpatient surge capacity and public health resilience.

In addition, this model holds promise beyond pandemic settings. Its modular informatics system, with real-time decision support, prescription automation, and health record access, can be adapted to scenarios like flu surges, natural disasters, or underserved areas. This approach offers a scalable blueprint for efficient outpatient care in crisis situations, though further research is needed to ensure that such operational gains are achieved without compromising care quality or patient safety.

## Figures and Tables

**Table 1:** Daily COVID-19 case numbers and field clinic visits in Taichung, 20 May–4 June 2022.

	Total		20–27 May (Before)		28 May–4 June (After)		*p* Value
	Mean	±SD	Mean	±SD	Mean	±SD	
Daily New COVID-19 Cases in Taichung City	9425	±1661	8932	±1518	9820	±1741	0.372
Patient Visits to Taichung Central Park Field Clinic	460	±144	469	±143	454	±152	0.762

Mann–Whitney U test.

**Table 2:** Baseline characteristics of patients before and after the implementation of the medical informatics system.

	Total (n = 8287)		20–27 May (Before) (n = 3749)		28 May–4 June (After) (n = 4538)		*p* Value
Age (mean ± SD)	39.73	±18.64	39.63	±18.98	39.81	±18.36	0.981
Male	4317	(52.09%)	1910	(50.95%)	2407	(53.04%)	0.058
Prescribed antiviral medication (Paxlovid or Molnupiravir)	1054	(12.72%)	361	(9.63%)	693	(15.27%)	<0.001 **
Paxlovid	983	(11.86%)	332	(8.86%)	651	(14.35%)	<0.001 **
Molnupiravir	71	(0.86%)	29	(0.77%)	42	(0.93%)	0.455
Physician seniority							<0.001 **
Attending physician	6602	(79.67%)	2463	(65.70%)	4139	(91.21%)	
Resident physician	1685	(20.33%)	1286	(34.30%)	399	(8.79%)	
Physician specialty							<0.001 **
Internal medicine	3097	(37.37%)	1478	(39.42%)	1619	(35.68%)	
Surgery	3102	(37.43%)	1165	(31.07%)	1937	(42.68%)	
Others	2088	(25.20%)	1106	(29.50%)	982	(21.64%)	

Chi-square test. ** *p* < 0.01. “Others” under physician specialty includes physicians from departments such as psychiatry, ophthalmology, obstetrics and gynecology, rehabilitation, anesthesiology, radiology, and radiation oncology.

**Table 3:** Comparison of physician consultation metrics before and after the implementation of the medical informatics system during peak clinic hours (10:00 AM–12:00 PM).

	20–27 May (Before)		28 May–4 June (After)		*p* Value
	Mean	±SD	Mean	±SD	
Number of physicians	11.38	±2.33	10.10	±3.60	0.653
Total number of consultations	138.63	±39.07	199.00	±37.60	0.001 **
Average number of consultations per physician	12.28	±3.08	22.51	±9.77	0.003 **
Maximum number of consultations per physician	38.25	±9.47	42.90	±8.82	0.370
Minimum number of consultations per physician	10.38	±8.47	21.20	±8.34	0.015 *
Number of antiviral prescriptions	19.00	±7.76	30.00	±12.14	0.046 *

Mann–Whitney U test. * *p* < 0.05, ** *p* < 0.01.

**Table 4:** Comparison of consultation metrics before and after the implementation of the medical informatics system, stratified by antiviral prescription status.

	Antiviral Prescribed					No Antiviral Prescribed				
	20–27 May (Before)		28 May–4 June (After)		*p* Value	20–27 May (Before)		28 May–4 June (After)		*p* Value
	Mean	±SD	Mean	±SD		Mean	±SD	Mean	±SD	
Number of physicians	4.63	±1.30	5.60	±0.84	0.102	5.75	±1.04	6.30	±0.95	0.227
Total number of consultations	19.00	±7.76	30.00	±12.14	0.046 *	119.63	±36.60	169.00	±30.88	0.007 **
Average number of consultations per physician	3.99	±1.28	5.25	±1.39	0.079	20.82	±5.22	27.18	±5.01	0.032 *
Maximum number of consultations per physician	7.13	±2.75	10.20	±4.66	0.122	33.50	±6.95	38.10	±9.62	0.444
Minimum number of consultations per physician	1.38	±0.52	2.00	±0.67	0.073	9.75	±8.03	16.30	±6.48	0.164

Mann–Whitney U test. * *p* < 0.05, ** *p* < 0.01.

## Data Availability

The datasets generated for this study are available on request to the corresponding authors.

## Supplementary Materials

The following supporting information can be downloaded at: https://www.mdpi.com/article/10.3390/healthcare13131514/s1, Table S1: CDC-defined risk factors for progression to moderate or severe COVID-19.

## Abbreviations

The following abbreviations are used in this manuscript:

COVID-19	Coronavirus Disease 2019
NHIA	National Health Insurance Administration
EHR	Electronic Health Record
PCR	Polymerase Chain Reaction
CDC	Centers for Disease Control and Prevention
