# Supplementary material for: Enhancing Consultation Efficiency Through Medical Informatics: A Scalable Field Clinic Model for the Pandemic Response in Taiwan
Source: Healthcare (Basel). 2025 Jun 25;13(13):1514. doi: 10.3390/healthcare13131514 (PMC12250337; doi:10.3390/healthcare13131514)
Supplement: Supplementary file 1 [file healthcare-13-01514-s001.zip › healthcare-3624852-supplementary.pdf]

Table S1. CDC-defined Risk Factors for Moderate to Severe Coronavirus Disease 2019 Outcomes

- Age ≥ 65
- Cancer
- Diabetes mellitus
- Chronic kidney disease
- Cardiovascular disease (not including hypertension alone)
- Chronic lung diseases (e.g., interstitial lung disease, pulmonary embolism, pulmonary hypertension, bronchiectasis, chronic obstructive pulmonary disease)
- Tuberculosis
- Chronic liver diseases (e.g., cirrhosis, non-alcoholic fatty liver disease, alcoholic liver disease, autoimmune hepatitis)
- Disabilities (e.g., attention-deficit/hyperactivity disorder, cerebral palsy, congenital malformations, intellectual and developmental disabilities, spinal cord injuries)
- Mental health disorders (e.g., mood disorders, schizophrenia spectrum disorders)
- Dementia
- Smoking (current and former)
- Obesity (body mass index ≥30 kg/m^2 or ≥95th percentile for teenagers/children)
- Pregnancy
- Immunodeficiency Conditions (e.g., human immunodeficiency virus, severe combined immunodeficiency, solid organ or hematopoietic cell transplantation, use of corticosteroids or other immunosuppressive medications)

Note: CDC = Centers for Disease Control and Prevention.
